# Association of the Dietary Index Underpinning the *Nutri-Score* Label with Oral Health: Preliminary Evidence from a Large, Population-Based Sample

**DOI:** 10.3390/nu11091998

**Published:** 2019-08-23

**Authors:** Valentina A. Andreeva, Manon Egnell, Pilar Galan, Gilles Feron, Serge Hercberg, Chantal Julia

**Affiliations:** 1 Equipe de Recherche en Epidémiologie Nutritionnelle (EREN), Centre de Recherche en Epidémiologie et Statistiques, Université Paris 13/INSERM U1153/INRA U1125/CNAM, F-93017 Bobigny, France; 2 Centre des Sciences du Goût et de l’Alimentation, AgroSup Dijon, CNRS, INRA U1324, Université Bourgogne Franche-Comté, F-21000 Dijon, France; 3 Département de Santé Publique, Hôpital Avicenne, F-93017 Bobigny, France

**Keywords:** diet quality, dietary index, nutrient profiling system, oral health, front-of-package label, public health

## Abstract

The 2017 implementation in France of the front-of-package nutrition label known as ‘Nutri-Score’ was intended as a public health strategy to help individuals make healthier food choices at the point of purchase and thus help reduce chronic disease on the population level. Nutri-Score and the associated individual-level dietary index are based on the British Food Standards Agency Nutrient Profiling System (FSAm-NPS-DI). Prior research has shed light on the relation between the dietary index and various physical health outcomes, yet no studies have explored the link with oral health. We analyzed the cross-sectional association of the dietary index with oral health in a population-based sample of 33,231 adults from the French NutriNet-Santé cohort. Oral health (main dependent variable) was assessed in 2016 with the General Oral Health Assessment Index; FSAm-NPS-DI (main independent variable) was calculated using ≥3 non-consecutive 24-h dietary records, following established methodology; lower scores corresponded to better diet quality. Age-specific associations were explored via multivariable linear regression. Fully-adjusted models showed modest yet significant associations between the dietary index and oral health in younger (18–59 years) and older (60+ years) participants, with the strength of the model being more pronounced in the former compared with the latter age group (F value: 28.5 versus 6.3, both *p* < 0.0001). Higher diet quality was associated with a somewhat lower risk of oral health problems. Albeit preliminary, the findings support the relevance of dietary indices underpinning nutrition labels such as the Nutri-Score. Future research is needed to confirm the associations.

## 1. Introduction

The World Health Organization (WHO) has called for individual, societal, and food industry-level measures to combat the alarming increase in the incidence and prevalence of chronic diseases stemming from the ever expanding consumption of fat/sugar-dense food coupled with sedentary lifestyles [1]. Individual-level recommendations pertain to limiting energy intake from fats/sugar, increasing intake of fruit/vegetables/legumes/nuts, and engaging in regular physical activity, whereas industry-level recommendations address reformulating food products in order to reduce fat/sugar/salt content of processed foods, and ensuring availability and affordability of nutritious food products [1]. In that context, the science of nutrient profiling has been gathering momentum via the development of algorithms for ranking food according to its nutritional composition. Front-of-package labels (FOPL) are one of the means for conveying such ranking information. Since the launch of the first FOPL (the Swedish “Keyhole symbol”) in 1989, various FOPL models have been developed and dozens of countries have been engaged in implementing FOPL on pre-packaged food/beverages [2]. The use of FOPL has been associated with a reduced intake of saturated fat, sugar, and total calories, an increased intake of fiber [3], and a reduced Body Mass Index (BMI) [4].

In response to these calls for the development of strategic public health tools to help consumers make healthier food choices and to trigger healthier food production [2], France implemented a five-color FOPL known as ‘Nutri-Score’ in 2017 [5]. The algorithm behind the Nutri-Score is intended to reflect overall nutritional quality and is largely based on the one developed by the British Food Standards Agency Nutrient Profiling System (FSA-NPS) [6,7], following a minor modification by the High Council of Public Health (FSAm-NPS) in conformance with French dietary guidelines [8]. This food-level index was subsequently used as the basis for an individual-level dietary index (FSAm-NPS-DI), described below. The FSAm-NPS-DI has been investigated with respect to risk of long-term weight gain [9], metabolic syndrome [10], cancer [11,12], and cardiovascular disease [13,14].

Some calls have been made for the development of FOPL models specifically targeting oral health [15]; however, current algorithms do not distinguish between general and oral health, given the number of common risk factors, such as increased intake of added sugars and decreased intake of fruit and vegetables [16]. In fact, WHO regards oral health—which reflects absence of pain, sores, gum disease, and tooth decay/loss—as key indicator of overall health, well-being, and quality of life (QOL) [16]. To our knowledge, no studies have investigated the link between any FOPL model or underlying score and oral health indices. Likewise, oral health research in non-elderly or non-clinical samples is scarce. The present study aimed to fill knowledge gaps by exploring the cross-sectional association of the dietary index with an oral health measure in a large, population-based sample.

## 2. Materials and Methods

### 2.1. NutriNet-Santé e-Cohort

We analyzed data from the NutriNet-Santé population-based cohort, launched in France in 2009 and currently ongoing (www.etude-nutrinet-sante.fr) [17]. The design, objectives, recruitment strategies, and methodology have been described elsewhere [17,18]. Briefly, men and women aged ≥18 years with Internet access are eligible for enrollment, and all assessment takes place online. NutriNet-Santé has received approval from the French Institute for Health and Medical Research ethics committee and from the National Commission on Informatics and Liberty. Following provision of informed consent and an electronic signature, each volunteer in the cohort was asked to complete a set of five baseline questionnaires: sociodemographics/lifestyle, dietary intake, physical activity, anthropometrics, and physical/mental health status. Thereafter, on a monthly or bi-monthly basis, self-report questionnaires on health- or nutrition-related topics were administered.

### 2.2. Oral Health Assessment

As part of follow-up, a questionnaire assessing oral health was administered between March and September 2016 (i.e., 5.6 ± 1.4 years after inclusion) to all active enrollees at the time (*n* = 120,448). It featured the General Oral Health Assessment Index (GOHAI), which is a self-report tool, initially designed to assess past-year oral health problems and oral health-related QOL of older adults, and later adapted for general use across age [19,20,21]. It consists of 12 items covering the domains of discomfort/pain and physical and psychosocial function. Each question is scored on a five-point Likert scale; thus, the maximum overall score is 60 points, with a higher score corresponding to better oral health. The following cutoff values have been established: total score ≤50 points, which reflects compromised oral health with a detrimental QOL impact; total score between 51 and 56 points, which reflects average oral health with some negative impact on QOL; score between 57 and 60 points, which reflects good oral health [19]. GOHAI has been translated and validated in French, demonstrating good psychometric properties, such as item-scale correlations between 0.40 and 0.78, and Cronbach’s alpha = 0.86 [20].

### 2.3. Dietary Index Calculation

Every 6 months, participants are asked to complete 3 24-hour dietary records by means of a user-friendly dietary record tool. Provided data should reflect dietary intake on three non-consecutive days during a pre-defined period of 2 weeks. Participants have at their disposal a search engine, a food and beverage browser, a user’s guide, and a built-in control system featuring cues and prompts designed to help maximize recall and minimize data entry errors. Participants are asked to report the portion size and/or quantity, recipe and/or seasoning, and corresponding setting (place and time) for each consumed food or beverage item. Portion sizes were estimated using validated photographs [22], and a food composition table featuring >3300 different items was used to estimate micro- and macro-nutrient intake [23]. The present analysis was based on dietary intake data from ≥3 non-consecutive 24-hour dietary records provided during the first two years following enrollment. For each participant, mean intake values were calculated across the available dietary records.

In order to obtain an individual-level dietary index (FSAm-NPS-DI), we first calculated the average nutrient profile at the food level and then aggregated those data at the individual level, following established methodology [24]. Specifically, per 100 g of each food/beverage consumed, positive points for “unfavorable” content were attributed, as follows: energy (kJ, 0–10 points), total sugar (g, 0–10 points), saturated fat (g, 0–10 points), and sodium (mg, 0–10 points); in turn, negative points for “favorable” content were attributed, as follows: proportion of fruit/vegetables/nuts (0–5 points), fiber (0–5 points), and protein (0–5 points). Thus, scores for food/beverages were represented on a discrete continuous scale ranging from −15 to +40, with lower scores corresponding to better overall diet quality (healthier nutrient profile) [24]. Next, aggregated scores at the individual level were calculated (i.e., FSAm-NPS-DI) using arithmetic energy-weighted means [24]. Higher FSAm-NPS-DI reflects lower nutritional quality of consumed food/beverages. The theoretical score range for the FSAm-NPS-DI is the same as the food-level FSAm-NPS.

### 2.4. Covariates

At inclusion, we collected data on sociodemographics (sex, age, height, weight, marital status, education, income, occupation) and lifestyle (smoking status, physical activity). Specifically, the International Physical Activity Questionnaire—Short Form was used to assess physical activity level according to established scoring criteria [25]. BMI was calculated as the weight (in kg) divided by the squared height (in m).

### 2.5. Statistical Analysis

In an effort to augment validity of the dietary intake data, we applied Black’s method [26] in order to identify dietary energy underreporting. That method accounts for age, sex, weight, height, physical activity level, and basal metabolic rate (calculated via Schofield’s equations) [27]. Thus, volunteers with likely or extreme energy underreporting were excluded from the analysis, as were those with <3 dietary records. Descriptive characteristics of the sample are reported as percentages from chi-squared tests or mean (SD) from Student t tests, as appropriate. The three-level GOHAI score (reverse-coded) was the main dependent variable; FSAm-NPS-DI, modeled on a continuous scale, was the main independent variable. Multivariable linear regression was used to estimate the cross-sectional associations. Model 1 was unadjusted; Model 2 was adjusted for sex and age (continuous variable); Model 3 was adjusted for sex, age (continuous variable), BMI (continuous variable), educational level (<high school; high school diploma or equivalent; some college/undergraduate degree; graduate degree), occupational status (homemaker/disabled/unemployed; student/trainee; blue collar/manual work; administrative/office staff; executive/professional staff; retired), marital status (living alone or married/cohabiting), monthly household income (seven-category variable), physical activity level (low, moderate, vigorous), smoking status (never, former or current smoker), and alcohol use (g ethanol/day, continuous variable). In line with evidence of sex-specific differences in FSAm-NPS-DI [24], we performed tests for interaction with sex. Additionally, because dietary intake and oral health are age-dependent [28], we added tests for interaction with age. A *p* < 0.05 (two-sided tests) was considered statistically significant. The statistical analysis was conducted with SAS (version 9.4, SAS Institute, Inc., Cary, NC, USA).

## 3. Results

### 3.1. Sample Characteristics

**Figure 1:**
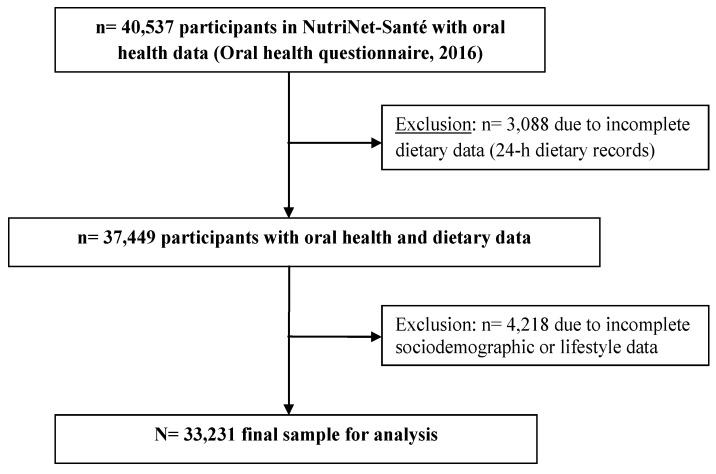
Participant selection flowchart.

Participant selection is summarized in Figure 1. From the sample of 40,542 volunteers who had completed the oral health questionnaire (and who were thus eligible for the present analysis), we excluded those with aberrant oral health data (*n* = 5), those with incomplete data for any of the sociodemographic or lifestyle variables (*n* = 4218), and those with incomplete or underreported dietary data, for whom the FSAm-NPS-DI could not be calculated (*n* = 3088). Thus, the final analysis sample included 33,231 participants.

Compared with those who were excluded from the analysis (*n* = 7306), included participants were somewhat more likely to be men, to be former or never smokers, married or cohabiting, and retired, and were slightly older (48.7 ± 13.8 years versus 44.5 ± 14.3 years) (all *p* < 0.0001; data not tabulated). Mean GOHAI score in the sample was 53.8 ± 5.5 (range: 19 to 60) and mean FSAm-NPS-DI was 6.0 ± 2.2 (range: −6.7 to +15.4). Tests for interaction with sex were not significant (*p* > 0.38), whereas significant interaction by age was observed (*p* < 0.01). Hence, age-specific analyses were performed using age = 60 years as cutoff, consistent with the definition of older age used in prior research employing GOHAI [29]. Descriptive characteristics of the sample are presented in Table 1.

Proportionally, older participants (ages 60+ years), compared with their younger counterparts (ages 18–59 years), included more men (40.3% versus 19.6%), fewer current smokers (6.3% versus 13.6%), and more individuals without secondary education (26.0% versus 12.8%); likewise, mean daily alcohol use appeared to be somewhat higher among older compared with younger participants (all *p* < 0.0001). Overall, the younger age group was more likely to report good oral health (40.3% versus 33.3%, *p* < 0.0001) yet had somewhat higher mean FSAm-NPS-DI, reflecting lower dietary quality (6.3 ± 2.3 versus 5.3 ± 1.9, *p* < 0.0001) compared with the older age group.

### 3.2. Association between FSAm-NPS-DI and Oral Health

Results of the age-specific linear regression analysis are presented in Table 2. The unadjusted (Model 1) and the partially adjusted model (Model 2, adjusted for sex and age) were significant only in the younger age group (ages 18–59 years). The fully adjusted model (Model 3, adjusted for sex, age, BMI, marital status, occupational status, education, household income, physical activity, smoking, and alcohol use) showed very modest yet significant associations in both age groups. The strength of the overall model was somewhat more pronounced in the younger compared with the older age group (F value: 28.5 versus 6.3, both *p* < 0.0001), as physical activity, marital status, and education were significant covariates only in the former group. Alcohol use was not a significant covariate in either age group.

## 4. Discussion

This study, carried out in a large, general population-based sample, provided some preliminary evidence of the link between a dietary index reflecting the overall nutritional quality of consumed food and oral health reflecting impact on QOL. The main finding was a modest, significant cross-sectional association between FSAm-NPS-DI and GOHAI, such that better overall diet quality was related to somewhat lower risk of oral health problems. To our knowledge, no prior research has been carried out in this domain. The analysis also provided some evidence of age-specific associations, suggesting that the link between diet quality and oral health might vary in strength and also with respect to the underlying determinants (covariates) between younger and older individuals. Given the relatively weak associations observed in this study, future cross-sectional as well as longitudinal analyses with more specific oral health measures are needed to replicate the models.

Several prior studies have modeled oral health as a predictor rather than outcome of dietary quality; poor oral health has been associated with reduced dietary variety, inadequate intake of micro- and macro-nutrients, increased risk of malnutrition, and BMI outside the normal range [30,31,32,33]. Edentulous individuals and those with impaired dentition were shown to consume fewer fruit/vegetables and less fiber, while having an increased intake of added sugars, saturated fat, cholesterol, and calories, and reduced concentrations of serum folate, beta carotene and vitamin C compared with their dentate counterparts [32,34,35,36,37]. Prior NutriNet-Santé research has also shown modest age-dependent inverse associations between oral health (measured with GOHAI) and diet quality (measured by adherence to French dietary guidelines) [28].

In our sample, GOHAI scores in younger and older participants were relatively high (thus, linear rather than logistic regression was the analysis of choice), while showing non-negligible variability at the individual level. GOHAI measures oral health with respect to its impact on QOL. It features items about physical function (concerns about swallowing, speaking, etc.), psychosocial function (concerns about self-image, avoiding social interactions, etc.), and discomfort/pain. It was selected as the outcome measure given its pertinence to an epidemiological context as well as its discriminant potential with respect to nutritional status [38]. Next, mean FSAm-NPS-DI values were similar to values reported in the EPIC [39] and SU.VI.MAX cohorts [9]. Prior validation research has shown that FSAm-NPS-DI was strongly related to micro- and macro-nutrient intake, nutritional status, and adherence to dietary guidelines [24]. The FSAm-NPS-DI is based on an energy- rather than quantity-weighted aggregation algorithm because the latter would likely result in reduced variability, as disproportionate weight would be attributed to food/beverages with high water content (i.e., 0 points) [24,40].

The FSAm-NPS belongs to the family of rating systems produced by nutrient profiling research. As with any such system, the ultimate test regarding its effectiveness is the ability to improve public health outcomes via improvements in food choices [41]. One of the earliest such systems was known as the Heart-Check Food Certification Program and symbol and was implemented by the American Heart Association in 1995 with the aim of helping consumers quickly and reliably identify foods that met the U.S. Food & Drug Administration requirements regarding benefits to cardiovascular health [41]. Consuming food marked with that symbol was associated with a reduced intake of added sugars, saturated fat, and sodium, and a reduced risk of obesity and metabolic syndrome [42]. Likewise, FSAm-NPS-DI has been inversely associated with risk of long-term weight gain [9], metabolic syndrome [10], cancer [11,12], and cardiovascular disease [13,14].

Given the interdependent and complex association among diet, nutrition, and oral health [43], the cross-sectional design, which prevented inference of causal links, is considered an important limitation of the study. Dietary and oral health data were not collected at exactly the same time point, with the former (exposure) generally preceding the latter (outcome). Next, GOHAI is an indirect measure of oral health and is not appropriate for dental disease diagnosis [19]. Moreover, it is often used in samples with compromised oral health [44,45,46]; thus, comparisons across studies might be challenging. Furthermore, our sample consisted of self-selected and likely health-conscious volunteers enrolled in a nutrition-focused cohort, which might explain their relatively good self-reported oral health status. It should also be noted that the majority of the working French population has access to affordable dental care, even though no organized oral health promotion strategies exist [47]. Additionally, given the relatively high GOHAI scores, the Internet-based design of NutriNet-Santé, and the fact that older age and retirement have been associated with reduced Internet access [48] and with increased risk of dental enamel erosion and oral health problems [49], we could speculate that the findings might be somewhat biased owing to the potentially low representation in the sample of retired individuals with compromised dental status. In turn, a limitation of any nutrient profile-derived algorithm, including the FSAm-NPS-DI, is that it entails a certain degree of subjectivity regarding selection of components, choice of cutoff values, and scoring criteria [50]. In addition, it is calculated on the basis of self-reported dietary data. Nonetheless, the algorithm is based on a previously validated model [6,7,51,52], and itself has undergone extensive validation [24,53]. Next, many volunteers were excluded from the analysis due to data completeness issues. Specifically, the study sample was composed of individuals who were somewhat more likely to be former/never smokers, slightly older, and married/cohabiting compared with their counterparts who were excluded from the analysis. These factors have been associated with oral health and/or diet quality [28,54] and therefore suggest the presence of potentially reduced variability in both the exposure and outcome measures. Moreover, the possibility of non-participation/selection bias necessitates caution when generalizing the findings. In turn, notable strengths of the study include its very large and heterogeneous sample of men and women recruited from the general population, the use of validated diet quality and oral health measures, and the inclusion of many potential confounders in the analysis.

## 5. Conclusions

With respect to disease prevention strategies at population level, oral health screening at midlife has been regarded as equally important as hypertension and hypercholesterolemia screening [55]. Disease burden regarding oral and other chronic diseases might be lessened by addressing common factors, such as reducing added sugar intake, striving for diet quality to prevent tooth decay, smoking cessation, limiting alcohol use, and maintaining oral hygiene [16]. The present study, to our knowledge, is the first to advance understanding regarding the link between an individual-level dietary index based on a FOPL model and oral health. Specifically, it provided preliminary support for an association between the nutritional quality of consumed food (measured via FSAm-NPS-DI) and oral health (measured via GOHAI). Future studies are needed to confirm these associations. On the public health policy level, the findings help support nutrition-oriented strategies, including implementation of FOPL models such as the Nutri-Score on pre-packaged food to steer consumer choice toward dietary behaviors that are favorable not only for chronic disease prevention but also for oral health status.

## Figures and Tables

**Table 1:** Descriptive characteristics of NutriNet-Santé participants by age group.

	Age 18–59 years (*n* = 24,243)	Age ≥60 years (*n* = 8988)	*P* ^1^
Sex			<0.0001
Male	4754 (19.61)	3626 (40.34)	
Female	19,489 (80.39)	5362 (59.66)	
FSAm-NPS-DI, mean (SD) ^2,3^	6.29 (2.25)	5.26 (1.92)	<0.0001
Dietary energy, Kcal/day, mean (SD) ^3^	1836.10 (475.70)	1833.00 (490.30)	0.60
Oral health, mean (SD) ^4^	54.03 (5.30)	53.03 (5.85)	<0.0001
Poor oral health, score ≤50	5100 (21.04)	2416 (26.88)	
Average oral health, score 51–56	9377 (38.68)	3578 (39.81)	
Good oral health, score 57–60	9766 (40.28)	2994 (33.31)	
Age, years, mean (SD)	42.84 (11.30)	64.59 (4.22)	<0.0001
Educational level			<0.0001
Less than high school	3091 (12.75)	2340 (26.03)	
High school diploma or equivalent	4261 (17.58)	2087 (23.22)	
Some college, undergraduate degree	7855 (32.40)	2196 (24.43)	
Graduate degree	9036 (37.27)	2365 (26.31)	
Occupational status			<0.0001
Homemaker/disabled/unemployed	3058 (12.61)	305 (3.39)	
Student/trainee	1332 (5.49)	0 (0.00)	
Manual/blue collar	5417 (22.34)	162 (1.80)	
Office work/administrative staff	5451 (22.48)	155 (1.72)	
Professional/executive staff	7457 (30.76)	457 (5.08)	
Retired	1528 (6.30)	7909 (88.00)	
Household income (monthly)			<0.0001
<1200 €	3591 (14.81)	466 (5.19)	
1200 €–1799 €	5881 (24.26)	1771 (19.70)	
1800 €–2299 €	3566 (14.71)	1438 (16.00)	
2300 €–2699 €	2149 (8.86)	1222 (13.60)	
2700 €–3699 €	3940 (16.25)	1803 (20.06)	
≥ 3700 €	2629 (10.84)	1494 (16.62)	
Not reported	2487 (10.26)	794 (8.83)	
Marital status			<0.0001
Married/cohabiting	17,862 (73.68)	6830 (75.99)	
Living alone (single, divorced, widowed)	6381 (26.32)	2158 (24.01)	
Body Mass Index, kg/m^2^, mean (SD)	23.71 (4.50)	24.91 (4.13)	<0.0001
Underweight (<18.5)	1361 (5.61)	211 (2.35)	
Normal weight (18.5–24.9)	15,783 (65.10)	4934 (54.90)	
Overweight (25.0–29.9)	4996 (20.61)	2909 (32.37)	
Obese (≥30.0)	2103 (8.67)	934 (10.39)	
Physical activity ^5^			<0.0001
Low	5443 (24.49)	1324 (15.54)	
Moderate	9680 (43.56)	3081 (36.16)	
Vigorous	7099 (31.95)	4115 (48.30)	
Smoking status			<0.0001
Never	12,949 (53.41)	4007 (44.58)	
Former	7999 (33.00)	4418 (49.15)	
Current	3295 (13.59)	563 (6.26)	
Alcohol use, g ethanol/d, mean (SD)	6.91 (10.42)	10.97 (13.40)	<0.0001

Values refer to number (%) except when noted otherwise. ^1^ *p*-values obtained from chi-squared tests or Student *t* tests, as appropriate; ^2^ FSAm-NPS-DI, Food Standards Agency modified Nutrient Profiling System-Dietary Index; score range between −15 and +40 points, with lower scores reflecting higher dietary quality; ^3^ Calculated from ≥3 24-hour dietary records provided during the first 2 years after enrollment; ^4^ General Oral Health Assessment Index (GOHAI), score range: 12 to 60 points, with lower scores reflecting worse oral health with a negative impact on QOL; ^5^ Assessed with the International Physical Activity Questionnaire-Short Form according to established scoring criteria, missing data from 2021 participants aged 18–59 years and 468 participants aged 60+ years. SD: standard deviation.

**Table 2:** Linear regression analysis of the age-specific association between the dietary index and oral health.

	Age 18–59 years (*n* = 24,243)			Age ≥60 years (*n* = 8988)		
	β	P(β)	F(model)	β	P(β)	F(model)
Model 1	−0.02	0.02	5.85	0.02	0.11	2.58
Model 2	0.02	0.005	112.59	0.02	0.06	11.49
Model 3	0.01	0.04	28.54	0.02	0.04	6.26

Linear regression analysis with FSAm-NPS-DI as the main exposure measure and the three-level GOHAI score as the main outcome measure; standardized ß. Model 1: unadjusted; Model 2: adjusted for sex and age; Model 3: adjusted for sex, age, marital status, educational level, occupational status, household income, BMI, physical activity, smoking status, and alcohol use.
